# Miliary Osteoma Cutis: A Case Report

**DOI:** 10.1155/2014/347829

**Published:** 2014-08-10

**Authors:** Felipe Aguinaga, Beatriz Trope, Juan Piñeiro-Maceira, Marcia Ramos-e-Silva

**Affiliations:** ^1^Sector of Dermatology and Post-Graduation Course, School of Medicine and University Hospital, Federal University of Rio de Janeiro, 21941-913, Rio de Janeiro, RJ, Brazil; ^2^Sector of Pathology and Post-Graduation Course in Dermatology, School of Medicine and University Hospital, Federal University of Rio de Janeiro, 21941-913, Rio de Janeiro, RJ, Brazil; ^3^Sector of Dermatology, Sector of Dermatology and Post-Graduation Course, School of Medicine and University Hospital, Federal University of Rio de Janeiro, Rua Dona Mariana 143/C-32, 22280-020 Botafogo, RJ, Brazil

## Abstract

The authors present a rare case of osteoma cutis miliaris and briefly update the current knowledge about its clinic, pathogenesis, and therapeutic options.

## 1. Introduction

Miliary osteoma cutis is a rare entity, with just over 50 cases described in the literature [1]. It is characterized by the emergence of multiple fragments of mature bone in the dermis.

Clinically, it presents as papules and normochromic hardened nodules similar to milia, which particularly affect the face, with a preference for women [1]. It may also occur as a late sequel in patients with acne vulgaris [2].

The authors report a rare case of miliary osteoma cutis and briefly comment on the current knowledge about its clinical presentation, pathogenesis, and therapy.

## 2. Case Report

A 62-year-old female patient complained about long standing lesions on the face. She denied any symptoms but wanted an aesthetic improvement of the lesions. She had a previous history of acne in adolescence without specific treatment.

**Figure 1:**
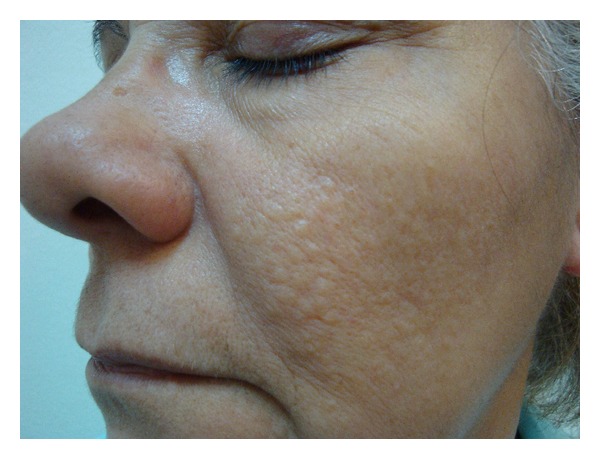
Normochromic grouped papules in the left malar region.

The examination showed several normochromic papules, hardened on palpation, grouped in the malar regions (Figure 1).

The very hard consistency of lesions caught our attention and this is why an ultrasound of the face was requested, which revealed small punctate echogenic spots with posterior acoustic shadowing, distributed diffusely through the dermis of the cheek, which could correspond to calcium deposits.

Laboratory tests revealed no changes in calcium metabolism or renal function.

**Figure 2:**
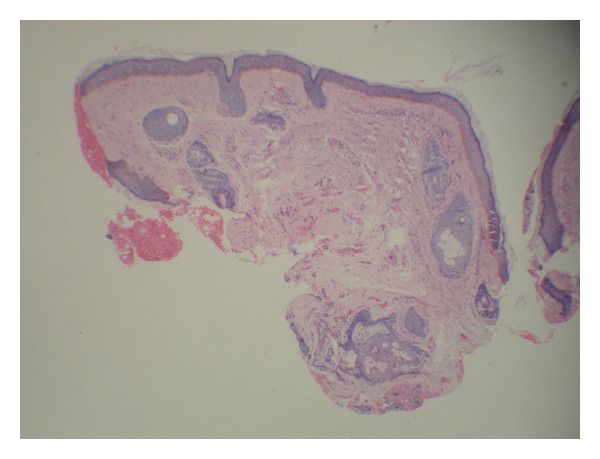
Histopathology showing central intradermal area of hyaline fibrosis and vascular neoproliferation where the osteoma became detached (HE, 100x).

**Figure 3:**
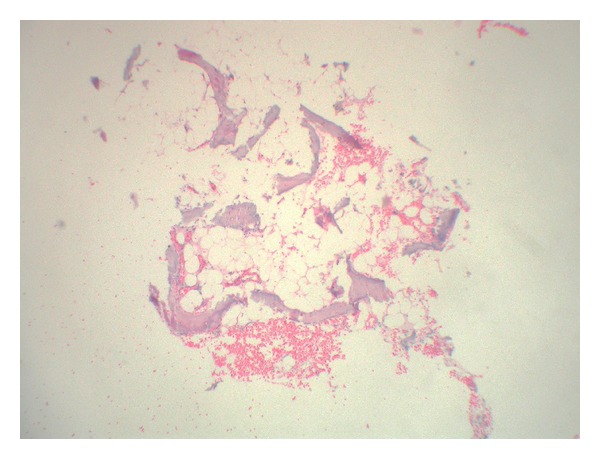
Mature bone fragment which became detached from the dermis during histologic processing, containing bone marrow (HE, 250x).

Histopathological examination showed a fragment of mature bone in the dermis, which became detached during the histologic processing, also containing bone marrow (Figures 2 and 3). Thus, the final diagnosis was miliary osteoma cutis.

As the patient refused to be submitted any surgical procedure, we chose to use retinoid acid cream at 0.1%. After 6 months of treatment, we did not notice any improvement in the number or appearance of the lesions. However, the patient was satisfied and believed that the lesions were less evident, and therefore did not want to undergo any complementary therapy.

## 3. Discussion

Cutaneous ossification stages are rare and are divided into primary and secondary. Rare genetic syndromes, often progressive and severe, are among the primary causes, each with phenotypic characteristics of their own, namely, progressive fibrodysplasia ossificans, progressive osseous heteroplasia, Albright's hereditary osteodystrophy, and plaque osteoma cutis [1–3].

Secondary or metaplastic causes constitute 80% of cases and appear as a consequence of previous lesions. Like cutaneous calcification, they may occur in collagen diseases, including dermatomyositis and scleroderma in panniculitis and in neoplasia, such as basal cell carcinoma and pilomatrichoma [1–3].

The classification of miliary osteoma cutis is controversial. Despite being traditionally described as a primary ossification form, it is associated with previous history of acne in 50% of cases [2], which would place it under both categories.

Miliary osteoma cutis was first described in 1864 by Virchow [1]. It is characterized by the emergence of papules and normochromic and hardened nodules, similar to milia. It affects mainly the face and has a predilection for women.

Hopkins, in 1928, was the first to suggest the role of acne in the development of multiple miliary osteoma cutis [2]. Patients who used tetracycline or minocycline may present bluish lesions [4].

Its pathogenesis is not entirely known. There are some hypotheses regarding the origin of the cell forming osteoma. The most accepted theory is fibroblast metaplasia [1, 2].* In situ* hybridization techniques have already shown that dermal fibroblasts have the ability to differentiate into osteoblasts and even to produce collagen type 1 and osteonectin [1].

Another hypothesis is that embryonic mesenchymal cells, erroneously migrated to the dermis, might differentiate into the osteogenic lineage [1]. Gene mutations involved in other syndromes of cutaneous ossification may also be implicated in the etiology [1].

There are several forms of treatment reported in the literature [3, 5, 6], including the use of retinoic acid at 0.1% [7], which is the option used by our patient, but the best results are obtained with surgical techniques, such as curettage [3] and needle excision [5], which are considered the treatment of choice.
